# *Abcc8* (sulfonylurea receptor-1) knockout mice exhibit reduced axonal injury, cytotoxic edema and cognitive dysfunction vs. wild-type in a cecal ligation and puncture model of sepsis

**DOI:** 10.1186/s12974-023-02692-2

**Published:** 2023-01-21

**Authors:** Jessica Cummings, Yijen L. Wu, C. Edward Dixon, Jeremy Henchir, J. Marc Simard, Ashok Panigrahy, Patrick M. Kochanek, Ruchira M. Jha, Rajesh K. Aneja

**Affiliations:** 1 Department of Critical Care Medicine, University of Pittsburgh, Pittsburgh, PA USA; 2 Department of Developmental Biology, University of Pittsburgh, Pittsburgh, PA USA; 3 Department of Neurosurgery, School of Medicine, University of Pittsburgh, Pittsburgh, PA USA; 4 Division of Pediatric Critical Care Medicine, Safar Center for Resuscitation Research, UPMC Children’s Hospital of Pittsburgh, University of Pittsburgh, Pittsburgh, PA USA; 5 Department of Neurosurgery, University of Maryland School of Medicine, Baltimore, MD USA; 6 Division of Pediatric Radiology, UPMC Children’s Hospital of Pittsburgh, Pittsburgh, PA USA; 7 Barrow Neurological Institute, Phoenix, AZ USA; 8 Department of Critical Care Medicine and Pediatrics, School of Medicine, Faculty Pavilion Building, University of Pittsburgh, 2nd Floor, Suite 2112, 4401 Penn Ave, Pittsburgh, PA 15224 USA

**Keywords:** Axonal injury, Cytotoxic edema, Microglial activation, Sepsis, Cecal ligation and puncture, Sulfonylurea receptor 1 (SUR1, *Abcc8*), Transient receptor potential melastatin 4 (TRPM4)

## Abstract

Sepsis-associated brain injury (SABI) is characterized by an acute deterioration of mental status resulting in cognitive impairment and acquisition of new and persistent functional limitations in sepsis survivors. Previously, we reported that septic mice had evidence of axonal injury, robust microglial activation, and cytotoxic edema in the cerebral cortex, thalamus, and hippocampus in the absence of blood–brain barrier disruption. A key conceptual advance in the field was identification of sulfonylurea receptor 1 (SUR1), a member of the adenosine triphosphate (ATP)-binding cassette protein superfamily, that associates with the transient receptor potential melastatin 4 (TRPM4) cation channel to play a crucial role in cerebral edema development. Therefore, we hypothesized that knockout (KO) of *Abcc8* (Sur1 gene) is associated with a decrease in microglial activation, cerebral edema, and improved neurobehavioral outcomes in a murine cecal ligation and puncture (CLP) model of sepsis. Sepsis was induced in 4–6-week-old *Abcc8* KO and wild-type (WT) littermate control male mice by CLP. We used immunohistochemistry to define neuropathology and microglial activation along with parallel studies using magnetic resonance imaging, focusing on cerebral edema on days 1 and 4 after CLP. *Abcc8* KO mice exhibited a decrease in axonal injury and cytotoxic edema vs. WT on day 1. *Abcc8* KO mice also had decreased microglial activation in the cerebral cortex vs. WT. These findings were associated with improved spatial memory on days 7–8 after CLP. Our study challenges a key concept in sepsis and suggests that brain injury may not occur merely as an extension of systemic inflammation. We advance the field further and demonstrate that deletion of the SUR1 gene ameliorates CNS pathobiology in sepsis including edema, axonal injury, neuroinflammation, and behavioral deficits. Benefits conferred by *Abcc8* KO in the murine CLP model warrant studies of pharmacological *Abcc8* inhibition as a new potential therapeutic strategy for SABI.

## Introduction

Sepsis-associated brain injury (SABI) imposes a substantial health burden on the survivors and their families [1–3] and is associated with an acute deterioration of mental status (septic encephalopathy), long-term persistent cognitive impairment with a subsequent reduction in the quality of life [1, 4–7]. Nearly 20,000 new cases per year of moderate to severe cognitive impairment in the elderly are potentially attributable to sepsis [1–4]. Historically, the brain was considered a privileged organ that is anatomically sequestered from the immune system by the blood–brain barrier (BBB) [8]. However, recent studies have suggested the presence of a functional lymphatic system in the central nervous system (CNS) engaged in constant immune surveillance [9] and that may play a role in SABI. With these new findings, we need to explore key pathomechanisms linked to immune activation in sepsis-induced brain injury.

To address this knowledge gap, we recently reported on the neuro-radiographic and histopathologic correlation of brain injury during the acute phase of sepsis in mice [10]. We noted that magnetic resonance imaging (MRI) of the brain in mice subjected to cecal ligation and puncture (CLP) revealed an early and marked decrease in fractional anisotropy (FA) consistent with axonal swelling and/or axonal injury in the cortex, hippocampus, and thalamus and significant decrease in apparent diffusion coefficient (ADC) values indicating restricted diffusion and cytotoxic edema. In addition, we demonstrated microglial activation in the same regions of interest (ROIs), suggesting that the findings on MRI were associated with neuroinflammation.

Recent studies in stroke and traumatic brain injury (TBI) have identified an important role for the sulfonylurea receptor1 (SUR1)-transient receptor potential melastatin-4 (TRPM4) ion channel in mediating the development of both cytotoxic and vasogenic edema post-insult and in the evolution of secondary brain injury [11–13]. SUR1 is a transmembrane receptor in the ATP binding cassette transporter family that is regulated after CNS injury to form an obligate association with Ca^2+^ sensitive TRPM4 resulting in oncotic edema (water influx via associated channels after injury) [12, 14–17]. Furthermore, gene suppression or pharmacological inhibition of *Abcc8* (SUR1 gene) ameliorates brain swelling and neuroinflammation with subsequent reduction in histological damage and improvement in neurological function in stroke and TBI [18–20]. The contribution of SUR1–TRPM4 in sepsis and its attendant CNS complications remain to be fully characterized. We hypothesized that knockout (KO) of *Abcc8* is associated with a decrease in microglial activation and brain edema and improved outcomes vs. wild-type (WT) in a murine CLP model of sepsis.

## Methods

### Murine model of sepsis

All the experiments in this report were approved by the University of Pittsburgh Institutional Animal Care and Use Committee. A total of 111 4–6-week-old male mice with global *Abcc8* KO on a C57/BL6J background were used with WT littermates as controls [21, 22]. Mice were housed with an inverse 12-h day–night cycle at 20–22 °C and had free access to food and water. Mice received general anesthesia with an inhaled 2% isoflurane/oxygen mixture. Body temperature was maintained using a circulating warming water block. CLP was performed as previously published [23, 24]. Briefly, a vertical midline incision was made through the abdominal wall, the cecum was externalized, and 60% was ligated with a 4–0 suture. The ligated cecum was punctured through-and-through one time with an 18-gauge needle. Two drops of fecal material were extruded, and the cecum was replaced into the peritoneal cavity. The abdominal incision was closed in 2 layers. Using the same general anesthesia and an identical protocol, sham mice underwent an abdominal incision followed by cecal extrusion and replacement without ligation or puncture. All mice received 20 ml/kg of normal saline subcutaneously (s.c.) after the procedure. Mice received enrofloxacin (5 mg/kg s.c.) twice daily and to ensure humane care, the long-acting analgesic buprenorphine (0.1 mg/kg s.c.) daily for the duration of the study. Each experimental group consisted of 4–8 mice.

### MRI and analysis

Mice were once again anesthetized using an inhaled 1–3% Isoflurane/oxygen mixture during the imaging session. They were placed into a clear plexiglass anesthesia induction box that allowed unimpeded visual monitoring of the animals. Induction was achieved by a brief administration of 3% Isoflurane mixed with oxygen. Depth of anesthesia was serially monitored by toe reflex (extension of limbs, spine positioning) and respiratory rate. Once the plane of anesthesia was established, it was maintained with 1–2% Isoflurane in oxygen via a nose cone and the mouse was transferred to a custom designed animal bed for imaging. The mouse head was secured and positioned with a bite bar in the nose cone and ear bars. Respiration was monitored using a pneumatic sensor placed between the animal bed and the mouse’s abdomen, while the rectal temperature was measured with a fiber optic sensor and maintained with a feedback-controlled warm air source to maintain normothermia (SA Instruments, Stony Brook, NY, USA).

#### In vivo MRI acquisition

*In-vivo* brain MRI was carried out on a Bruker BioSpec 70/30 USR spectrometer (Bruker BioSpin MRI, Billerica, MA, USA) operating at 7-Tesla (T) field strength, equipped with an actively shielded B-GA12S2 gradient with 440 mT/m gradient strength and slew rate 3440 T/m/s. The MRI was acquired with an 2X2 mouse brain array receiver surface coil in conjunction with a 72 mm volume coil for radiofrequency (RF) transmission.

#### *T*2*-weighted (T2W) anatomical imaging*

Multi-planar T2W anatomical imaging was acquired with Rapid Imaging with Refocused Echoes (RARE) pulse sequence with the following parameters: field of view (FOV) = 1.7 cm, matrix = 256 X 256, slice thickness = 1.5 mm, in-plane resolution = 66 µm X 66 µm, RARE factor = 8, effective echo time (TE) = 48 ms, repetition time (TR) = 1500 ms, flip angle (FA) = 180 degrees.

#### *T*2 *mapping*

Multi-planar quantitative T2 relaxometry was performed with Hahn spin echo imaging using 8 echo times (10.49, 20.97, 31.46, 41.95, 52.44, 62.92, 73.41, 83.90 ms), TR = 4000 ms, and the same imaging voxel dimension as the T2WT images. Least squares analysis based on a single exponential decay were performed using Bruker XTip software to obtain the quantitative T2 maps.

#### Dynamic contrast enhancement (DCE) and T1-weighted (T1W) MRI for BBB integrity

To acquire dynamic contrast-enhanced (DCE) images, multi-planar T1W images obtained with the Fast Low Angle Shot (FLASH) pulse sequence with the same imaging voxel dimension as the T2W imaging with the following parameters: TE = 2.94 ms, TR = 200 ms, FA = 30 degrees. Three baseline images were obtained before contrast administration. A single bolus of gadolinium contrast (0.2 mmol/kg in 100uL; MultiHance®, Bracco Diagnostic Inc., Cranbury, NJ) was administered via intraperitoneal injection and 7–8 post-contrast T1W images were obtained.

#### Diffusion MRI

Multi-planar diffusion MRI was acquired with the following parameters: field of view (FOV) = 1.7 cm, matrix = 128 X 128, slice thickness = 1.5 mm, in-plane resolution = 132.8 µm X 132.8 µm, TE = 12.31 ms, TR = 1100 ms, diffusion preparation with the stimulated echo sequence, diffusion gradient duration = 2 ms, diffusion gradient separation = 60 ms, diffusion direction = 12, number of A_0_ image = 1, and b value = 1500 s/mm^2^. Diffusion MRI was analyzed by the open source DSI studio (http://dsi-studio.labsolver.org/) to obtain fractional anisotropy (FA), ADC, axial diffusivity (AD), and radial diffusivity (RD) maps. The ROIs, including cortex, hippocampus, thalamus, corpus callosum, and ventricles with cerebrospinal fluid, were manually segmented and defined by blinded independent obversions for quantitative and statistical analysis.

T1W signal intensities were measured using Paravision 5.1 (Bruker, Billerica, MA). The ROIs were manually outlined on a T2W image of a representative sham animal and superimposed on all corresponding T1W images. For pre-contrast images, the mean signal intensity (SI) value for each ROI was averaged across the 3 acquired images. For post-contrast images, the mean SI for each ROI was measured at each timepoint and averaged across animals in the same group. The % change in T1W SI between the pre- and post-contrast images was calculated for each timepoint. The test was performed by a technician who was blinded to experimental design.

### IHC and analysis

While under deep terminal anesthesia, mice were perfused transcardially with phosphate-buffered saline (PBS). Brains were extracted, fixed in 10% formalin overnight, and submerged in 70% ethanol for at least 24 h. Brains were frozen in liquid nitrogen-cooled 2-methylbutane and stored in − 80ºC. Brains were processed in paraffin and sections (5 µm) of brain tissue spanning bregma coordinates − 2.12 to − 2.75 were immunostained and analyzed.

Sections were stained with anti-ionized calcium-binding adaptor molecule-1(Iba-1) antibody (Wako Chemicals Inc., Richmond, VA) and Vectastain ABC kit (Vector Laboratories, Burlingame, CA) according to the manufacturers’ instructions [25]. Briefly, sections were deparaffinized and placed in 2% H_2_O_2_ for 20 min and blocked with 3% normal goat serum (Vector, Burlingame, CA) in TBST for 30 min. Sections were incubated overnight in goat serum/TBST solution containing rabbit anti-Iba-1 antibody (1:200, Wako Chemicals, Osaka, Japan). The sections were then incubated for 1 h with anti-rabbit HRP-conjugated secondary antibody (ABC Elite Kit, Vector) and stained with 3,3'-diaminobenzidine (DAB, Vector). Sections were examined under 20× magnification using a Nikon 90i microscope and analyzed with NIS Elements Advanced Research software (Nikon Instruments Inc., Melville, NY). Photomicrographs were obtained from 3 randomly selected, nonadjacent areas in each ROI. Iba-1^+^ cells were counted from each area and averaged to determine the number of microglia/mm^2^.

### Assessment of long-term behavioral and neuropathological outcomes over 14 days

A total of 28 mice were studied in this protocol. To determine if motor or cognitive performance was altered in *Abcc8* KO vs. WT mice, in this protocol, we evaluated the performance of both sham and septic mice on these behavioral tasks. Mice were thus randomly divided into four groups WT (sham), *Abcc* KO (sham), WT (CLP) and *Abcc8* KO (CLP), *n* = 8 per CLP group, and *n* = 6 per sham group, respectively.

### Assessment of motor function and Morris water maze (MWM) performance

Analysis of motor function and MWM spatial memory acquisition was performed as previously described [26, 27]. WT and *Abcc8* KO mice underwent CLP as described above (*n* = 10 in each group). For sham animals, testing of WT and KO in the behavioral protocols (as described below) temporally matched the approach taken for the CLP group. Motor function was assessed at days 1–5 after CLP using a beam balance task, with mice trained on day 0 (pre-injury). Spatial memory acquisition was assessed using the MWM hidden platform task on days 7–11 followed by probe trial. Performance in the MWM was quantified by measuring latency in finding the hidden platform and the probe trial was quantified as percent time in the target quadrant when the platform is removed. Visible platform testing was then performed on days 14 after CLP to confirm that latencies were not confounded by visual impairments. Data were analyzed by a trained technician who was blinded to experimental conditions.

### Statistical analysis

All data are presented as means ± SEM unless otherwise stated. After exclusion of normal distribution using the Shapiro–Wilks test, differences between all groups were analyzed with a one-way Kruskal–Wallis analysis of variance on ranks followed by a post hoc Dunn’s test for multiple comparisons. A *P* value < 0.05 was considered statistically significant.

## Results

### *Abcc8* KO reduces MRI markers of cytotoxic edema vs. WT after CLP

**Fig. 1:**
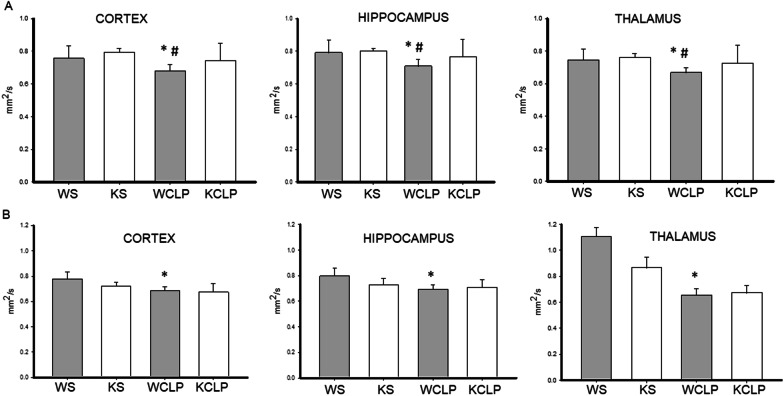
Effect of *Abcc8* knockout (KO) on apparent diffusion coefficient (ADC) in brain after cecal ligation and puncture (CLP) in mice, as assessed by magnetic resonance imaging (MRI). On day 1 (**A**) and day 4 (**B**), after CLP, wild-type (WT) and *Abcc8* KO mice underwent brain MRI (WT CLP (WCLP), KO CLP (KCLP)). Additional controls included shams (WT SHAM (WS), KO SHAM (KS)). Regions of interest (ROI) analysis was performed after manual delineation of a priori regions of the brain that include the cortex, hippocampus, and thalamus. Mean ADC values are depicted. Each graph represents the mean ADC value from a specific ROI. (*P < 0.05 vs. WS; ^#^ *P* < 0.05 vs. KCLP) *P* values were calculated by ANOVA, and error bars represent SEM) (sham *n* = 5, and CLP *n* = 10 for each timepoint)

Previously, we reported the absence of vasogenic edema and BBB disruption in our murine CLP model as evidenced by T2WT anatomical images and T1-weighted MRI post-Gadolinium contrast images [10]. In the current study, at 1 day after CLP, we noted significant cytotoxic edema as evidenced by a decrease in the ADC in WT mice in the cortex, hippocampus, or thalamus vs. sham WT mice, respectively (0.67 ± 0.04 vs. 0.75 ± 0.06 mm^2^/s; *P* = 0.003; 0.70 ± 0.04 vs. 0.79 ± 0.08 mm^2^/s; *P* = 0.01; 0.66 ± 0.03 vs. 0.74 ± 0.07 mm^2^/s; *P* = 0.018) (Fig. 1A). However, compared to sham, *Abcc8* KO mice subjected to CLP did not demonstrate a significant decrease in the ADC for cortex, hippocampus and thalamus indicating a reduction in diffusion restriction or cytotoxic edema (0.79 ± 0.02 vs. 0.74 ± 0.08 mm^2^/s; *P* = 1.00; 0.79 ± 0.02 vs. 0.76 ± 0.08 mm^2^/s; *P* = 1.0; 0.76 ± 0.02 vs. 0.72 ± 0.08 mm^2^/s; *P* = 1.0). Furthermore, ADC values were significantly lower for WT vs. *Abcc8* KO mice after CLP for the three ROIs (0.67 ± 0.04 vs. 0.74 ± 0.08 mm^2^/s; *P* = 0.009; 0.70 ± 0.04 vs. 0.76 ± 0.08 mm^2^/s; *P* = 0.03; 0.66 ± 0.03 vs. 0.72 ± 0.08 mm^2^/s; *P* = 0.01).

However, by day 4 after CLP, there was no difference in the ADC values in all three ROIs in *Abcc8* KO mice vs. WT (0.68 ± 0.03 vs. 0.67 ± 0.07 mm^2^/s; *P* = 1.00; 0.69 ± 0.02 vs. 0.70 ± 0.06 mm^2^/s; *P* = 1.0; 0.65 ± 0.03 vs. 0.67 ± 0.05 mm^2^/s; *P* = 1.0) (Fig. 1B). Therefore, compared to WT, *Abcc8* KO was associated with a reduction in acute cytotoxic edema and cellular swelling in mice that were subjected to CLP.

### *Abcc8* KO reduces MRI markers of axonal injury vs WT after CLP

**Fig. 2:**
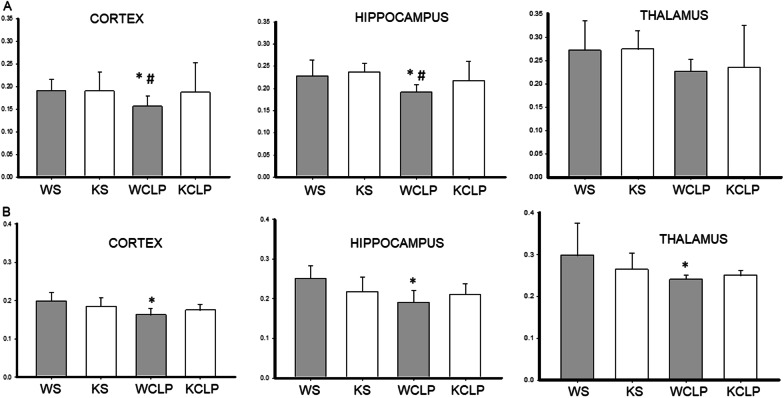
Effect of *Abcc8* knockout (KO) on fractional anisotropy (FA) in brain after cecal ligation and puncture (CLP) in mice, as assessed by magnetic resonance imaging (MRI). On day 1 (**A**) and day 4 (**B**) after CLP, wild-type (WT) and *Abcc8* KO mice underwent brain MRI (WT CLP (WCLP), KO CLP (KCLP)). Additional controls included shams (WT SHAM (WS), KO SHAM (KS)). Regions of interest (ROI) analysis was performed after manual delineation of a priori regions of the brain that include the cortex, hippocampus, and thalamus. Mean FA values are depicted in this figure. Each graph represents the mean FA value from a specific ROI. (*P < 0.05 vs. WS; ^#^ *P* < 0.05 vs. KCLP) *P* values were calculated by ANOVA, and error bars represent SEM) (sham *n* = 5, and CLP *n* = 10 for each timepoint)

The FA values (to measure axonal integrity) were significantly different between sham and WT mice subjected to CLP on day 1 in two ROIs (cortex: 0.19 ± 0.02 vs. 0.15 ± 0.01; *P* = 0.03; hippocampus: 0.22 ± 0.03 vs. 0.19 ± 0.01; *P* = 0.03), respectively (Fig. 2A). FA values were not significantly different in the thalamus (0.27 ± 0.06 vs. 0.22 ± 0.02; *P* = 0.53). The FA values in the *Abcc8* KO mice subjected to CLP were comparable to the sham mice and significantly higher than the WT mice in the two ROIs mentioned above (cortex: 0.18 ± 0.04 vs. 0.15 ± 0.01; *P* = 0.04; hippocampus: 0.21 ± 0.03 vs. 0.19 ± 0.01; *P* = 0.01). As with ADC values, by day 4 after CLP, the mean FA values in the cortex, hippocampus, and thalamus did not differ between the *Abcc8* and WT mice (cortex: 0.17 ± 0.01 vs. 0.16 ± 0.01; *P* = 0.27; hippocampus: 0.20 ± 0.03 vs. 0.19 ± 0.03; *P* = 0.32, and thalamus: 0.25 ± 0.01 vs. 0.24 ± 0.01; *P* = 1.0), respectively (Fig. 2B).

**Fig. 3:**
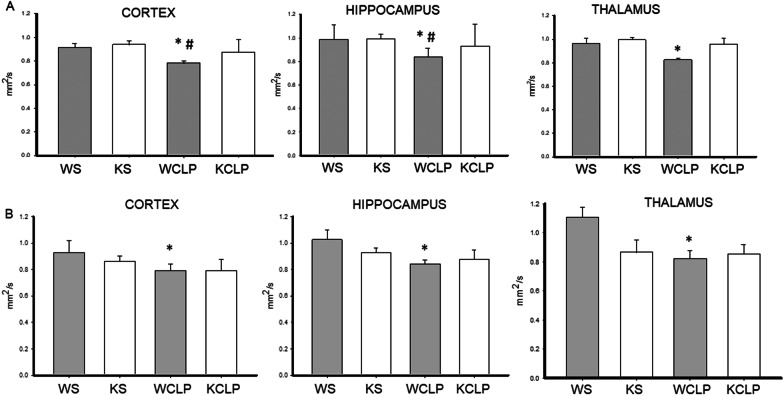
Effect of *Abcc8* knockout (KO) on axial diffusivity (AD) in brain after cecal ligation and puncture (CLP) in mice, as assessed by magnetic resonance imaging (MRI). On day 1 (**A**) and day 4 (**B**) after CLP, wild-type (WT) and *Abcc8* KO mice underwent brain MRI (WT CLP (WCLP), KO CLP (KCLP)). Additional controls included shams (WT SHAM (WS), KO SHAM (KS)). Regions of interest (ROI) analysis was performed after manual delineation of a priori regions of the brain that include the cortex, hippocampus, and thalamus. Mean AD scalars are shown. Each graph represents the mean AD value from a specific ROI. (*P < 0.05 vs. WS; ^#^ *P* < 0.05 vs. KCLP) *P* values were calculated by ANOVA, and error bars represent SEM) (sham *n* = 5, and CLP *n* = 10 for each timepoint)

To examine the possible microstructural changes, we calculated mean tract-averaged AD and RD values for the ROIs, as AD and RD are the most important indices associated with FA [28]. We noted a significant decrease in AD in WT mice subjected to CLP on day 1 vs. sham in the three ROIs (cortex: 0.78 ± 0.05 vs. 0.91 ± 0.09 mm^2^/s; *P* = 0.004; hippocampus: 0.83 ± 0.06 vs. 0.98 ± 0.14 mm^2^/s; *P* = 0.01; thalamus: 0.82 ± 0.05 vs. 0.96 ± 0.14 mm^2^/s; *P* = 0.015). AD values in the *Abcc8* KO mice subjected to CLP were significantly higher than WT in two ROIs suggesting a reduction in axonal injury in *Abcc8* KO mice vs. WT (cortex: 0.87 ± 0.10 vs. 0.78 ± 0.05 mm^2^/s; *P* = 0.008; hippocampus: 0.92 ± 0.13 vs. 0.83 ± 0.06 mm^2^/s; *P* = 0.018) (Fig. 3A). No significant changes in RD were noted between sham, WT, and *Abcc8* KO mice subjected to CLP (data not shown). By day 4, although significant differences were noted between sham and WT mice, AD values were not different between WT and *Abcc8* KO mice subjected to CLP (Fig. 3B). Summarizing, the acute AD values in *Abcc8* KO mice in the cortex and the hippocampus were significantly higher than the WT mice after CLP suggesting a reduction in axonal injury in the *Abcc8* KO mice. Taken together, our data suggest that *Abcc8* KO mice demonstrated a reduction in acute cytotoxic edema (cellular swelling) and axonal injury vs. WT mice after CLP.

### ***Abcc8*** KO mice exhibit reduced microglial activation vs. WT early after CLP

**Fig. 4:**
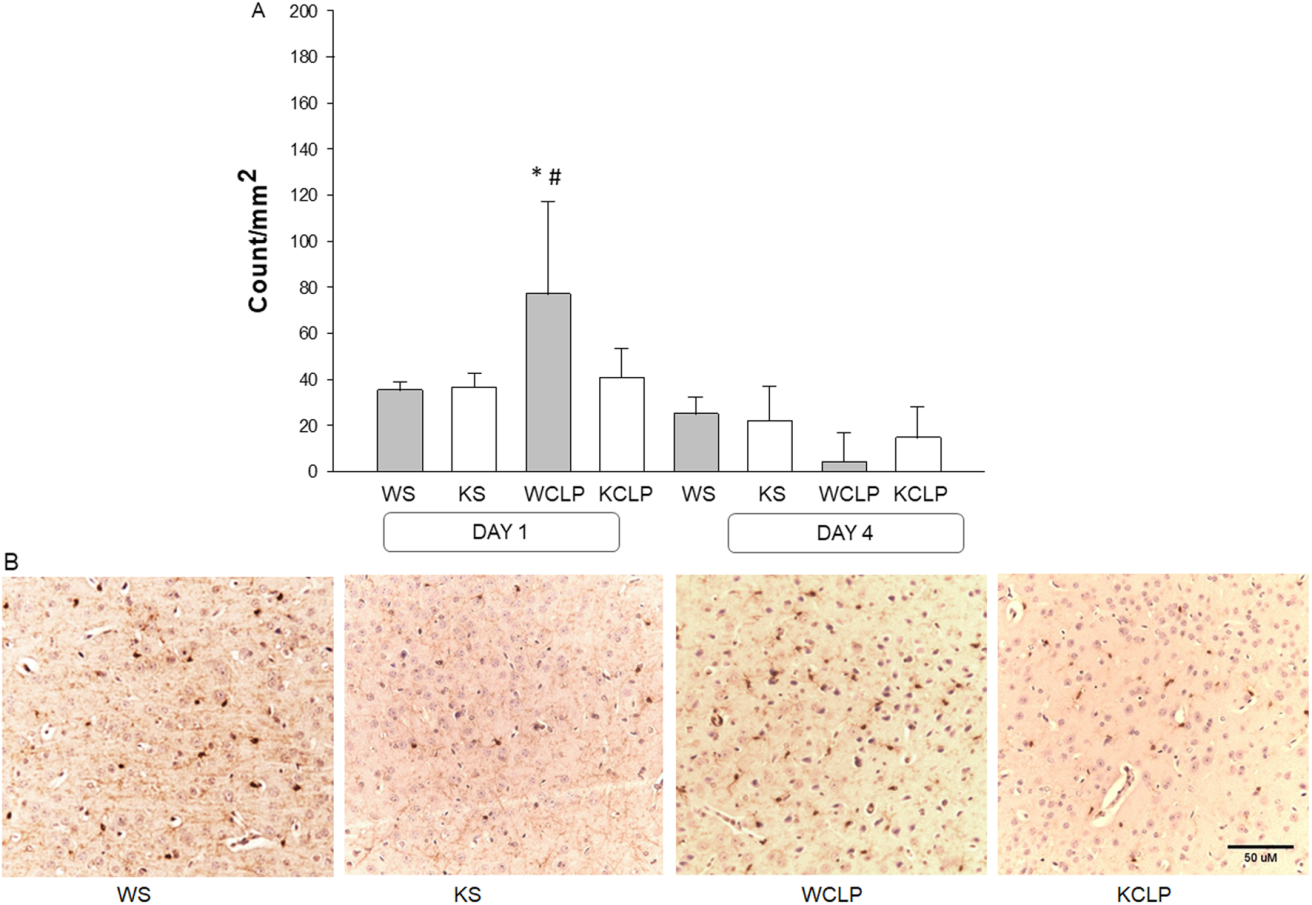
Effect of *Abcc8* knockout (KO) on the microglial response in brain after cecal ligation and puncture (CLP) in mice, as assessed using ionized calcium-binding adaptor molecule-1 (Iba-1) immunohistochemistry. Representative micrographs of brain areas stained for Iba-1 in the regions of interest (ROI) of sham and CLP mice on day 1 after CLP. The quantification of activated microglia is shown in **A**. Illustrative micrographs demonstrate a significant increase in Iba-1 + microglial cells on day 1 after CLP (20 × magnification) in the cortex that was not seen in the *Abcc8* KO (**B**). (*P < 0.05 vs. WS; ^#^ *P* < 0.05 vs. KCLP) *P* values were calculated by ANOVA, and error bars represent SEM) (sham *n* = 10, and CLP *n* = 15 for each timepoint)

To understand the potential role of *Abcc8* in CLP-mediated neuroinflammation and its subsequent impact on axonal injury and cytotoxic edema, we quantified cortical microglia (Iba1^+^) numbers in the same ROIs assessed using MRI in the WT and *Abcc8* KO septic mice. The number of Iba-1^+^ cells was significantly higher on day 1 in the cortex of WT CLP mice vs. sham mice (76.96 ± 33.3 vs. 35.3 ± 7.2 cells/mm^2^, *P* < 0.001). In contrast, the number of Iba-1^+^ cells were comparable in the CLP induced *Abcc8* KO mice and sham (40.5 ± 13.0 vs. 36.4 ± 8.6 cells/mm^2^, *P* = 1.0); *Abcc8* KO mice demonstrated attenuated microglial activation in the cortex vs. WT mice after CLP (40.5 ± 13.0 cells/mm^2^ vs. 76.96 ± 33.3 *P* < 0.001) (Fig. 4A, B). The number of microglia did not significantly differ in the other two ROIs in *Abcc8* KO vs. WT mice. By day 4, there was no difference in Iba-1^+^ cells in the cortex between shams, WT, and *Abcc8* KO mice subjected to CLP (Fig. 4A, B).

Taken together, these results indicate significant cytotoxic edema in septic WT mice in the cortex, hippocampus, or thalamus vs. sham WT mice on day 1 which resolved by day 4 after CLP. *Abcc8* KO mice subjected to CLP demonstrate attenuation of acute cytotoxic edema, axonal injury, and neuroinflammation as reflected by microglial activation on day 1 vs. WT mice.

### *Abcc8* KO mice exhibit reduced behavioral deficits vs WT after CLP

**Fig. 5:**
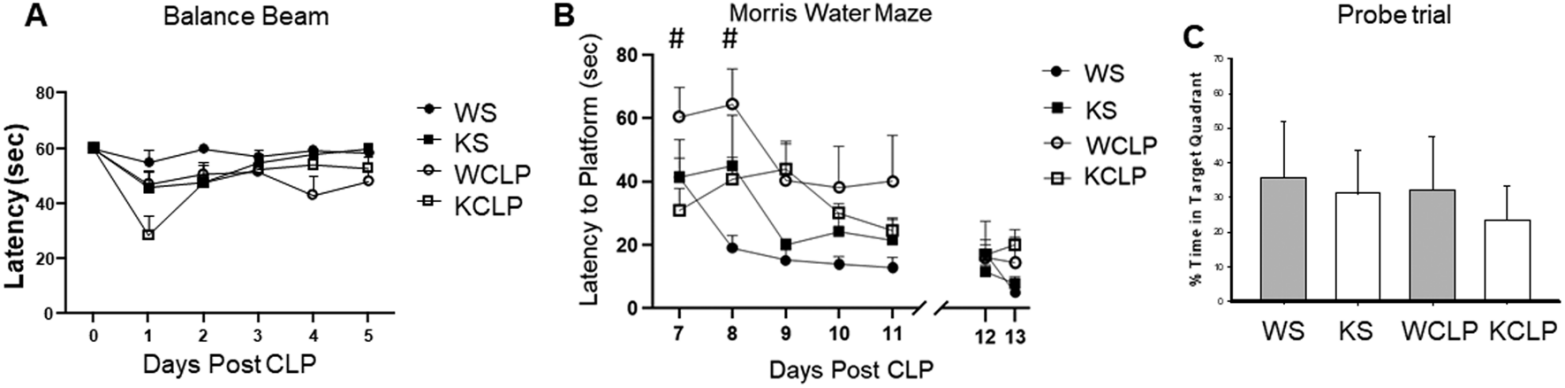
Behavioral outcomes after cecal ligation and puncture (CLP), in wild-type (WT) and *Abcc8* knockout (KO) mice as assessed by (**A**) beam balance test (**B**) spatial memory acquisition in the Morris water maze (MWM) paradigm and (**C**) probe trail. **A** *Abcc8* KO mice exhibit a nonsignificant decrease in latency on the beam balance task on day 1 post-CLP and improved on days 2–5 after CLP (*P* > *0.05*). **B** WT mice demonstrated significant impairment in learning on the MWM 7–8 day post-CLP (^#^*P* < 0.05 vs. KCLP). A visible platform task was performed on days 12–13 post-CLP. **C** Probe trial was performed on days 12–13 post-CLP and % time in target quadrant was comparable in all groups (sham *n* = 6, and CLP *n* = 8/genotype)

Both WT and *Abcc8* KO mice exhibited a non-significant decrease in the latencies on the beam balance task on day 1 after CLP vs. sham mice. The latencies gradually improved for both groups on days 2–5 after CLP, and there was no significant difference in the overall performance between the septic WT and *Abcc8* KO mice (Fig. 5).

Spatial memory acquisition was tested using the MWM on days 7–11 after CLP and on identical days in the sham mice. No differences were noted in the submerged platform latencies for sham *Abcc8* KO vs. WT mice on days 7–11 of testing (Fig. 5). The deletion of *Abcc8* was associated with a significant decrease in latency to platform on days 7–8 of the MWM in mice subjected to CLP vs. WT mice (*P* < 0.05). No differences were noted in the submerged platform latencies for *Abcc8* KO vs. WT mice on days 9–11 of testing (Fig. 5).

To discriminate between spatial and non-spatial strategies, mice were given a probe test on day 12 after CLP (Fig. 5). The % time in the target quadrant was similar between the sham WT and *Abcc8* KO groups (35.5 ± 0.1% vs. 34.5 ± 6.0; *P* = 0.276). Similarly, the % time in the target quadrant in WT and *Abcc8* KO mice was not significantly different after CLP (32.17 ± 16.0% vs. 23.3 ± 7.0%, respectively). Visible platform latencies for both sham mice and mice after CLP were similar and are shown in Fig. 5. Swim speeds were equal between groups indicating no motor impairment during spatial memory testing (data not shown). In summary, our data suggest that cognitive function is transiently impaired after CLP in both genotypes, but the impairment is less pronounced in the *Abcc8* KO vs. WT mice.

## Discussion 

Previously, we reported that septic mice had evidence of axonal injury, robust microglial activation, and cytotoxic edema in the cortex, thalamus, and hippocampus in the absence of BBB disruption vs. sham controls after CLP [10]. Upregulation of SUR1 has been shown to mediate cytotoxic and vasogenic edema in mechanical trauma to the CNS [12] including TBI [29], spinal cord injury [30], and subarachnoid hemorrhage [19]. Studies in TBI and stroke indicate that post-injury edema can be reduced and outcomes improved by either pharmacological inhibition of *Abcc8* or via KO of the *Abcc8* gene [11, 12, 21]. Although much of the published data relate to TBI and stroke, seminal work by Simard et al. showed upregulation of Sur1–Trpm4 channels in adult rats who received lipopolysaccharide (LPS) infusion into the striatum [31]. Therefore, we hypothesized that genetic deletion of SUR1–TRPM4 would be associated with a decrease in microglial activation, edema, and improved outcomes in our murine CLP model of sepsis. In this study, we showed that in comparison with WT, the *Abcc8* KO mice subjected to CLP showed (1) reduced acute cytotoxic edema and axonal injury in several brain regions, (2) decreased microglial activation in the cortex, and 3) reduced neurocognitive impairment after CLP.

Intracellular ATP depletion activates SUR1–TRPM4 with cell depolarization leading to an influx of Na^+^ with the obligatory secondary movement of Cl^−^ producing the driving force for water movement and subsequent cytotoxic edema [12, 15, 18, 29, 32]. In our murine model of sepsis, the WT CLP mice demonstrated a significant reduction in the ADC values indicating restricted mobility of water molecules in the cortex, thalamus, and hippocampus signifying cytotoxic edema. In contrast, the absence of *Abcc8* was associated with a significant reduction in cytotoxic edema in all three ROIs and improved spatial learning vs. WT septic mice.

Based on our MRI data, the *Abcc8* pathway appears to play a role 24 h after the induction of CLP. Whether administering GLY within the first 24 h after CLP could confer benefits remains to be defined, given that our studies were limited to the evaluation of this pathway in mice with constitutive KO of the *Abcc8* gene. Nevertheless, this strategy of using GLY has shown promise in multiple pre-clinical studies in stroke and TBI [10–12] and has been reported to improve neuronal preservation and neurological outcome in stroke [33] and malignant cerebral edema models [15, 33]. Furthermore, GLY has also shown benefit on secondary outcomes in an initial clinical trial in stroke [34] and is currently being tested in a phase-2 clinical trial in TBI (ASTRAL, NCT03954041) [11].

Despite the success of SUR1–TRPM4 inhibition in stroke and TBI models, we must be mindful of the pathophysiology unique to SABI. It has been shown that local acidosis in the brain can enhance GLY-mediated SUR1–TRPM4 inhibition, which may play a role in the importance of this pathway in injured brain regions in stroke and TBI. However, the impact of acidosis on this pathway in sepsis remains to be defined [35]. Similarly, the variability of the BBB impairment in sepsis merits careful evaluation as GLY does not penetrate the intact BBB. Studies of the effect of GLY in our CLP model are ongoing.

Identifying SUR1–TRPM4 as a crucial mediator of cerebral edema was a seminal event in our understanding of CNS injury [11]. Using *Abcc8* KO mice, we were able to advance the field by defining the contribution of SUR1–TRPM4 to the cytotoxic edema and neuroinflammation that occurs after sepsis. Together, these studies challenge a crucial concept in sepsis: *brain injury is an extension of systemic inflammation*. Instead, our work, combined with the work of Simard et al. [31], suggests that upregulation of the SUR1–TRPM4 channel in the brain directly contributes to the CNS pathobiology previously demonstrated [36].

FA measures the degree of directionality of diffusion, and a reduction was observed in the hippocampus, thalamus, and cortex. We considered potential causes associated with a significant decrease in FA, including myelin sheath damage, changes in axonal diameter or axonal packing density, and increased membrane permeability [37–39]. To this end, we noted a significant alteration in AD, which is associated with axon morphological changes, i.e., changes in axonal density or caliber [28], suggesting acute axonal swelling and/or axonal injury on day 1. We did not notice a significant increase or decrease in RD, suggesting demyelination or increased myelination that could contribute to a change in FA [40]. Similar changes in AD and FA have been reported in an inflammatory axonal loss in multiple sclerosis [41, 42], and mild TBI [43–45]. The above-noted acute changes in AD and FA resolved by day 4 after CLP suggesting that these changes are transient and resolve once the acute inflammatory response wanes. There is potential for clinical relevance here, given that recent studies of septic patients have demonstrated both serum biomarker evidence of axonal injury, notably increases in neurofilament light, along with MRI evidence [46, 47]. Finally, recent studies in TBI have suggested significant genetic variability in *Abcc8*–TRPM4 between patients that is strongly associated with its role in mediating secondary damage [48, 49]. This suggests the possibility of precision-directed therapy, thereby warranting further investigation into the genetic underpinning of this pathway in patients with septic encephalopathy [21, 49].

In vivo and in vitro studies on microglia showed that activation of TLR4 by LPS led to de novo upregulation of SUR1–TRPM4 channels and calcineurin dephosphorylating nuclear factor of activated T cells (NFAT)-dependent upregulation of nitric oxide synthase mRNA and protein [31]. We investigated microglial changes in the areas interrogated by MRI and noted a spatial association between microglial activation and subsequent axonal injury and cytotoxic edema in the cortex on day 1 after CLP. A reduction in the expression of pro-inflammatory cytokines in models of subarachnoid hemorrhage and experimental autoimmune encephalomyelitis was associated with both pharmacological inhibition of *Abcc8* or *Abcc8* gene suppression [18, 20, 50]. Our data are congruent with these observations that have examined the deletion of *Abcc8* in TBI and experimental autoimmune encephalomyelitis─i.e., reduced inflammation and axonal preservation.

We reported less robust associations in the thalamus and hippocampus, which could be related to varying effects of the *Abcc8* pathway in different brain regions, sampling issues, or variability in the model. Although the research personnel were independent for the MRI and the behavioral experiments, the laboratory personnel could not be blinded to experimental details for the microglia staining and counting. This was due to limited resources during the pandemic. Still, the appropriate control groups establish a sufficient degree of rigor for this experiment. Our results also contrast with Fukushima et al., who demonstrated that a single injection of LPS increased microglial proliferation in the fornix and dentate gyrus but not the cerebral cortex and corpus callosum of adult mice [50]. However, LPS injection and CLP reflect related albeit different insults. Microglia are a family of diverse phenotype cells [51, 52], that may have different proliferative capability depending on the insult and brain region. The proliferation of microglia is gated by the inflammatory response which is contingent on the distributing patterns between the cerebral vasculature and different brain regions. The quantitative distributing patterns between the vasculature and specific brain regions in the mouse have not been reported previously [53], but some progress in this field has been made recently [54]. Future research addressing the mechanisms underlying these regional differences in microglia activation during systemic inflammation are warranted.

Our study is also novel as we have included MWM performance in the CLP model, and to our knowledge, very little data exist regarding MWM performance after CLP. The MWM testing started on post-CLP day 7 to explore the possible impact of the early pathophysiologic role of *Abcc8* on the development of cognitive deficits. Given the need to ensure that swim speed is not impaired for this task and assess the early behavioral deficits in the mice, the earliest we could perform MWM was day 7. WT CLP mice performed worse than the sham WT mice. Since there were no significant differences between the other groups, this indicates that injured *Abcc8* KO was associated with improved MWM performance compared to the sham Abcc8 mice. Additional studies of cognitive outcomes using MWM in the CLP model are warranted, including differences in both timing of assessment and insult severity, to optimize its use.

Taken together, our work and the work of other investigators suggest that further study is needed to understand the role of inflammation in the development of axonal injury and brain edema in sepsis, both in pre-clinical models and at the bedside. Our studies also suggest an excellent translational opportunity to understand the pathobiology and develop therapies to mitigate the long-term consequences.

There are several limitations to this study. First, we used a rigorous approach with pre-determined ROIs, which may have inhibited discovery in other regions. Furthermore, a much more comprehensive assessment of microglial phenotype could help define detrimental vs. beneficial effects of neuroinflammation in our model [55, 56]. Second, our study was conducted only on male mice. Although there is a growing interest in understanding the effect of gender on the immune response following inflammatory conditions, i.e., hemorrhage and sepsis [57], there is no study that we could find that has characterized the effect of gender on SABI. Preclinical studies suggest that low dihydrotestosterone and/or high estradiol may protect the host, as evidenced by survival and systemic inflammatory response [57–60]. Thus, although female sex hormones exhibit protective effects under septic conditions, it is unclear if this extends to the brain in sepsis. In contrast, Tata et al*.* recently showed that TBI produced a greater degree of cerebral atrophy in WT male vs. female mice, and loss of *Abcc8* vigorously protected against post-traumatic cerebral atrophy only in males [21]. Potential sex differences in the role of the SUR1–TRPM4 pathway in SABI remain to be explored. Third, in gene KO cell lines or tissues, unexpected compensatory or redundant mechanisms develop in response to the missing gene and can confound experimental observations. To ascertain if the benefits noted in the CLP model are due to *Abcc8* inhibition, we need to verify them using inducible KO mice or with chemical inhibition using GLY—although off target effects of GLY could play a role [11, 21]. Fourth, further studies are needed to define the role of aquaporin-4 or other channels for water movement that underlie the development of radiographically detectable cytotoxic cerebral edema early after CLP. This is particularly important given that aquaporin-4 channels play a role in water movement triggered by cation accumulation through *Abcc8*–TRPM4 [11].

In summary, deletion of Abcc8 (*Abcc8* gene) was associated with an attenuation of axonal swelling, cytotoxic edema, and neuroinflammation in a murine CLP model of sepsis, which was associated with improved cognitive performance. Our data suggest that inhibition of *Abcc8* is a promising strategy that should be explored pre-clinically to define its potential for clinical translation to prevent or treat SABI.

## Data Availability

The data sets used and/or analyzed during the current study are available from the corresponding author on reasonable request.
